# Preface to 'Nurturing resilient marine ecosystems'

**DOI:** 10.1098/rstb.2022.0135

**Published:** 2022-07-04

**Authors:** Dame Linda Partridge

**Affiliations:** Vice President and Biological Secretary, The Royal Society, 6 Carlton House Terrace, London SW1Y 5AG, UK

Biodiversity loss and threats to the world's ecosystems represent some of the most critical challenges facing humanity today. The 2019 IPBES report found that nature is declining globally at unprecedented rates in human history, with around 1 million plant and animal species now threatened with extinction. Marine ecosystems, from coastal to deep sea, now consistently show the influence of human actions, and live coral cover on reefs has almost halved in the past 150 years. Insights from science are essential to better understanding these trajectories, including the impacts of climate change and human activity, as well as the actions that will help to halt and reverse these trends.

With this in mind, the Royal Society, in partnership with the African Academy of Sciences, chose ‘Science for a resilient future’ as the theme for the third Commonwealth Science Conference, which was held virtually in February 2021 and brought together 350 scientists (of whom 270 were early-career) from 32 Commonwealth nations. The conference focused on three key themes: (1) *Developing resilient energy systems*; (2) *Nurturing resilient ecosystems*; and (3) *Building resilient societal systems.* This special issue of *Philosophical Transactions* *B* focuses primarily on the second theme, although with some discussion of topics relevant to the third. The first theme has been addressed in a separate issue of *Philosophical Transactions A*.

The Conference came at a critical moment, ahead of two major UN conferences on climate change (COP26) and biodiversity (COP15) and the Commonwealth Heads of Government Meeting, at the start of the UN Decade of Ocean Science for Sustainable Development and UN Decade of Ecosystem Restoration, and in the context of the recently published Dasgupta Review on the Economics of Biodiversity. Although commissioned by the UK Government, the Dasgupta Review contains messages of global importance, including that human demands on nature are vastly outweighing nature's capacity to provide the goods and services we depend on, and that we need to rethink our measures of economic success in order to tread a more sustainable path.

Recognizing this timely opportunity, alongside the conference I have been leading on behalf of the Royal Society a work programme to amass a substantial package of evidence on biodiversity and to raise its prominence in policy thinking and action. Three major themes have emerged from this work: the need for nature to be valued and accounted for in decision-making, including in economic policy; the need to find cross-sectoral solutions to biodiversity loss, climate change and development, rather than looking at biodiversity in isolation; and the importance of developing a global biodiversity monitoring network. These are matters of global importance and I hope this special issue will help to stimulate and develop international collaborations in this important field.

I would like to express my thanks to the Steering Committee of the Commonwealth Science Conference, to all the speakers and participants at the conference and to the staff of the Royal Society and African Academy of Sciences for their work in organizing the conference sessions on which this special issue is based. The conference was funded by the UK Government Global Challenges Research Fund (GCRF).

